# Automatic hand phantom map generation and detection using decomposition support vector machines

**DOI:** 10.1186/s12938-018-0502-8

**Published:** 2018-06-11

**Authors:** Huaiqi Huang, Claudio Bruschini, Christian Antfolk, Christian Enz, Tao Li, Jörn Justiz, Volker M. Koch

**Affiliations:** 1 BME Lab, Institute for Human Centered Engineering, Bern University of Applied Sciences, Quellgasse 21, 2502 Biel, Switzerland; 2 Integrated Circuits Laboratory (ICLAB), École Polytechnique Fédérale de Lausanne (EPFL), Rue de la Maladiére 71b, 2002 Neuchâtel, Switzerland; 3 Biomedical Engineering, Lund University, Ole Römers väg 3, 22100 Lund, Sweden

**Keywords:** Phantom map, Support vector machines, Sensory feedback, Hand amputee, Machine learning, Active learning

## Abstract

**Background:**

There is a need for providing sensory feedback for myoelectric prosthesis users. Providing tactile feedback can improve object manipulation abilities, enhance the perceptual embodiment of myoelectric prostheses and help reduce phantom limb pain. Many amputees have referred sensation from their missing hand on their residual limbs (phantom maps). This skin area can serve as a target for providing amputees with non-invasive tactile sensory feedback. One of the challenges of providing sensory feedback on the phantom map is to define the accurate boundary of each phantom digit because the phantom map distribution varies from person to person.

**Methods:**

In this paper, automatic phantom map detection methods based on four decomposition support vector machine algorithms and three sampling methods are proposed, complemented by fuzzy logic and active learning strategies. The algorithms and methods are tested on two databases: the first one includes 400 generated phantom maps, whereby the phantom map generation algorithm was based on our observation of the phantom maps to ensure smooth phantom digit edges, variety, and representativeness. The second database includes five reported phantom map images and transformations thereof. The accuracy and training/ classification time of each algorithm using a dense stimulation array (with 100 $\times$ 100 actuators) and two coarse stimulation arrays (with 3 $\times$ 5 and 4 $\times$ 6 actuators) are presented and compared.

**Results:**

Both generated and reported phantom map images share the same trends. Majority-pooling sampling effectively increases the training size, albeit introducing some noise, and thus produces the smallest error rates among the three proposed sampling methods. For different decomposition architectures, one-vs-one reduces unclassified regions and in general has higher classification accuracy than the other architectures. By introducing fuzzy logic to bias the penalty parameter, the influence of pooling-induced noise is reduced. Moreover, active learning with different strategies was also tested and shown to improve the accuracy by introducing more representative training samples. Overall, dense arrays employing one-vs-one fuzzy support vector machines with majority-pooling sampling have the smallest average absolute error rate (8.78% for generated phantom maps and 11.5% for reported and transformed phantom map images). The detection accuracy of coarse arrays was found to be significantly lower than for dense array.

**Conclusions:**

The results demonstrate the effectiveness of support vector machines using a dense array in detecting refined phantom map shapes, whereas coarse arrays are unsuitable for this task. We therefore propose a two-step approach, using first a non-wearable dense array to detect an accurate phantom map shape, then to apply a wearable coarse stimulation array customized according to the detection results. The proposed methodology can be used as a tool for helping haptic feedback designers and for tracking the evolvement of phantom maps.

## Background

Although the dexterity of hand prostheses has made significant progress in the past decades, there is still no or limited sensory feedback in commercial prostheses [1, 2]. To our knowledge, the only commercial prosthesis equipped with sensory feedback is the Vincent Evolution 2 hand from the Vincent Systems GmbH [3]. This hand has only one vibrator, providing very limited feedback. A survey conducted by Pylatiuk et al. showed that upper arm amputees would like to have sensory feedback integrated in the hand prosthesis [4]. Providing sensory feedback can not only improve the functionality of the prosthesis, but also enhance the body ownership feeling of the amputees. Another benefit is to relieve phantom limb pain [5].

There are several ways to provide amputees with tactile sensory feedback. The methods can be roughly divided into two main categories: invasive and non-invasive feedback. The invasive approach stimulates the central nervous system using cortical electrodes [6] or the peripheral nervous system using either cuff electrodes [7, 8] or transversal intrafascicular multichannel electrodes [9]. Non-invasive feedback systems apply stimuli on the surface of the skin. The stimuli can be electrical currents (electrotactile) [10–14], vibrations (vibrotactile) [11] or mechanical pushing (mechanotactile) [15, 16] on the skin to elicit sensations.

**Fig. 1:**
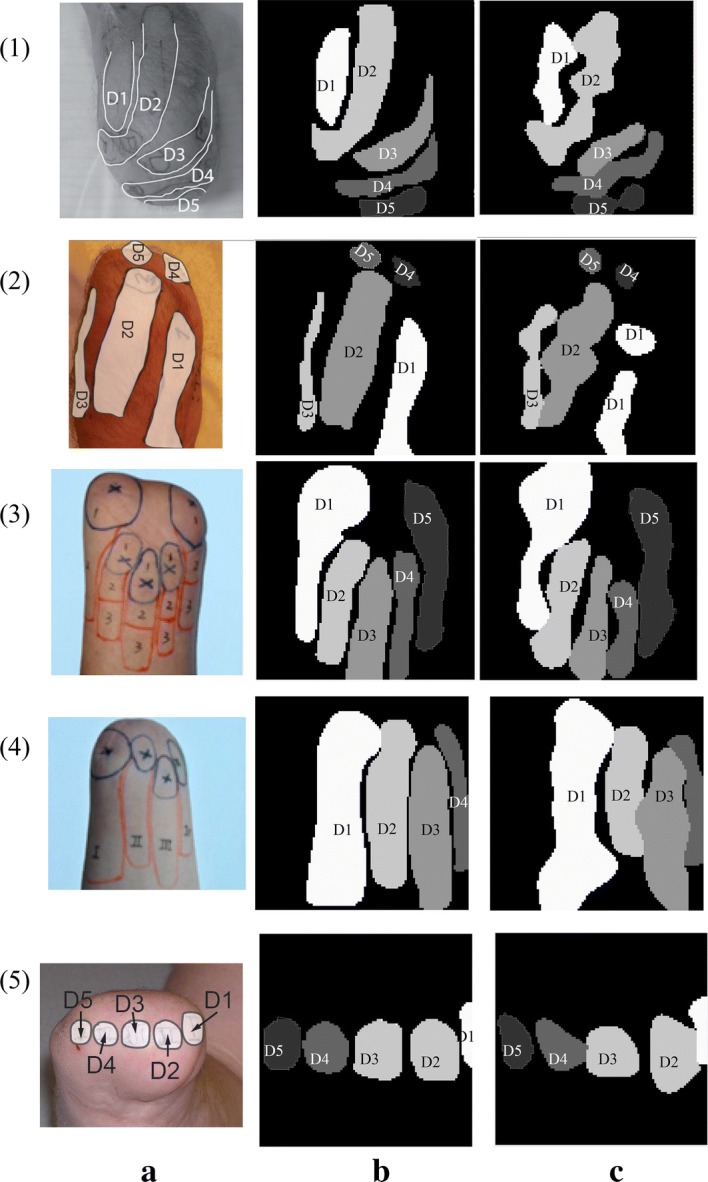
Reported phantom map image classification examples. Examples of **a** reported phantom map images [13, 23, 27, 28], **b** processed and down-sampled phantom maps, and **c** predicted phantom maps using OVO-SVM and $2 \times 2$ majority pooling

Many amputees have referred sensation of their lost arm on their remaining stump, called phantom map (examples of phantom maps are shown in Fig 1a). Phantom map could serve as an area to provide sensory feedback. A phantom map is a region on the body that can evoke a sensation of the lost hand. Surveys have shown that 80–90% of amputees develop a phantom map immediately after amputation [17]. While half of those amputees keep stable long-term phantom maps [17], most of the time, the hand phantom maps are present on the face or on the remaining stump. Pressure applied on one area of the phantom map gives the sensation that it comes from a specific finger or an area of the amputated hand.

The dominant theory regarding the phantom map formation is the reorganization of the cortical topography. In the Penfield map, the hand area is bordered by the upper arm and the face. When the hand is amputated, these two regions (upper arm and face) invade the area representing the hand, thereby forming the phantom map [18].

Previous works have demonstrated the feasibility of providing non-invasive sensory feedback on the hand phantom maps [19–21]. One of the benefits of providing sensory feedback on the phantom map is its high spatial resolution. We have indeed observed that the phantom map area has a smaller two-point discrimination distance than the contralateral upper arm, for example. It has also been reported that somatosensory feedback (feedback applied on the phantom map) has a higher discrimination accuracy and requires lower mental workload when the amputees are required to recognize the stimulation pattern [19].

The phantom map distribution and sensitivity vary from individual to individual because of the “uncontrollable muscle and nerve reorganization” which takes place after the amputation [17, 19]. The phantom map shape can also change over time [18].

Finding the hand phantom map distributions of individual amputees is of great importance in order to provide efficient stimulation patterns and take full advantage of the high spatial resolution provided by the phantom map.

Current phantom map detection methods are still quite rudimentary, using palpation and then drawing the phantom map directly on the skin of the amputee [22, 23]. This detection method is inconsistent and inaccurate. It is difficult to record detailed phantom map distribution and to track the evolvement of each individual phantom map. Moreover, to the best of our knowledge, no phantom map database exists at present.

Current sensory feedback applied on phantom maps normally uses one actuator (vibrator or pusher) per phantom finger [20]. In this approach, a rough phantom map distribution detection is sufficient. However, when a dense stimulation array is applied on the phantom map, a more accurate phantom map distribution estimation is needed. The WiseSkin project, for example, aims at providing richer feedback to upper arm amputees by incorporating dense, miniaturized sensory nodes in a hand glove, and using wireless transmission to convey the sensory data to a processing unit [24]. The latter generates signals to activate a corresponding dense actuator array placed on the phantom map of the amputee.

## Previous work

**Fig. 2:**
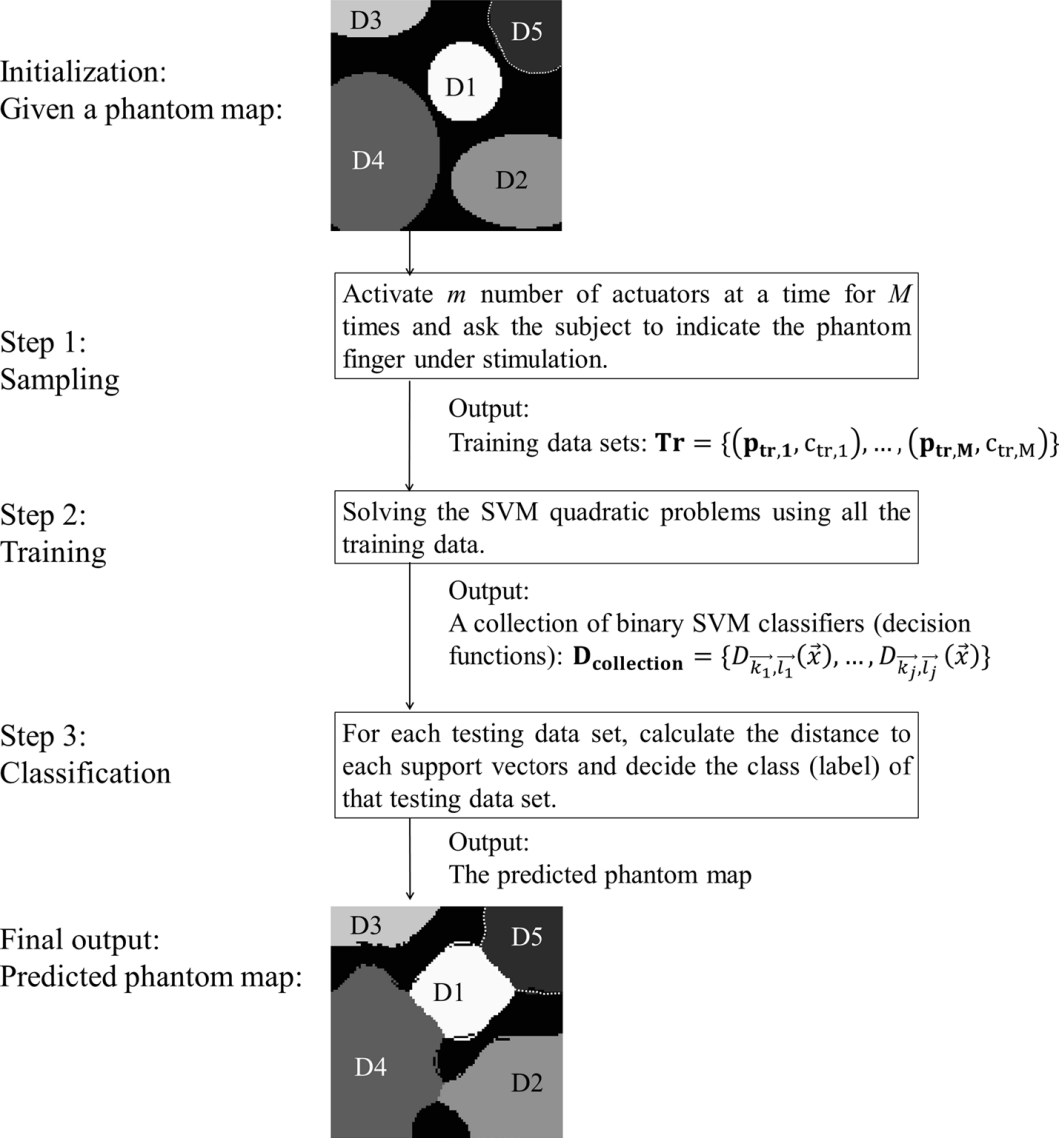
Automatic phantom map detection flow. Automatic phantom map detection flow diagram. In the flow graph, only the generated phantom map images are used as an example

An automatic phantom map detection method has been proposed in our previous work [24, 25]. The proposed method collects a small amount of phantom map distribution data of an amputee and uses this data and different algorithms to generate a detailed graphical phantom map. An example of automatic phantom map detection using SVM algorithms is shown in Fig. 2. This approach, which was tested on simulated phantom map models, can help feedback designers to customize the stimulation array and potentially increase the haptic vocabulary. It can also help to find an optimized place for electromyography (EMG) electrodes to avoid interactions between EMG electrodes and sensory feedback arrays. The proposed algorithms included two non-machine learning based (group testing and adaptive edge finding [24]) and two machine learning based algorithms (neural network [24] and support vector machine (SVM) [25]). The simulation results showed that support vector machine based algorithms have higher overall accuracy than the other tested algorithms. They were however based on simplified ellipse-based phantom map models and only their overall error rates were compared, without a detailed training and classification cost analysis (i.e. analysis of the algorithm sensitivity for a series of figures of merit, and training or classification time).

The general approach in this paper is similar to the flow in previous work (Fig. 2), consisting of three stages: sampling, training, and classification. However, this paper substantially extends the previous work in all stages and proposes four multi-class SVM algorithms for automatic phantom map detection, complemented by fuzzy SVM and active learning to further increase the detection accuracy. More realistic and detailed contour phantom map models were generated to test the algorithms using the specifications of fine grained stimulation arrays. Refined metrics were used to evaluate the effectiveness of the algorithms, including on the performance of a “realistic” coarse stimulation array. A time cost and shifting error analysis was included as well.

This paper is organized as follows: the hand phantom map databases, including a novel contour phantom map model generation and reported phantom map processing, are introduced in “Hand phantom map databases” section, then three data sampling methods are presented in “Sampling methods” section. The proposed support vector machine algorithms with and without fuzzy membership functions or active learning are detailed in “Support vector machine” section. The accuracy and timing aspects of the proposed algorithms are presented “Results” section and the results are discussed in “Discussion” section. Conclusions are provided in “Conclusion” section.

## Hand phantom map databases

Two databases are introduced in this section to test the phantom map detection algorithms: the first database consists of generated phantom maps using a contour model (“Hand phantom map model generation” section), whereas the second database is based on processed and transformed reported phantom map images (“Database from reported phantom map images” section).

### Hand phantom map model generation

In this subsection, we describe the generation process which we employed to produce realistic phantom maps using a contour model (Fig. 2: Initialization). The distribution and sensitivity of phantom maps do vary individually. From the descriptions of the reported phantom maps and interviews with amputees, it can for example be observed that phantom maps have clear and smooth edges [19, 23]. There can be repeated phantom digit representations (one phantom finger has more than one non-connected area represented on the phantom map) [19]. Some amputees have a complete phantom map, meaning that all five phantom fingers exist, while others only have partial phantom maps (one or more phantom fingers are missing). Furthermore, it is also observed that when several phantom digits are touched simultaneously, the amputee can distinguish all the digits that are being touched.

**Fig. 3:**
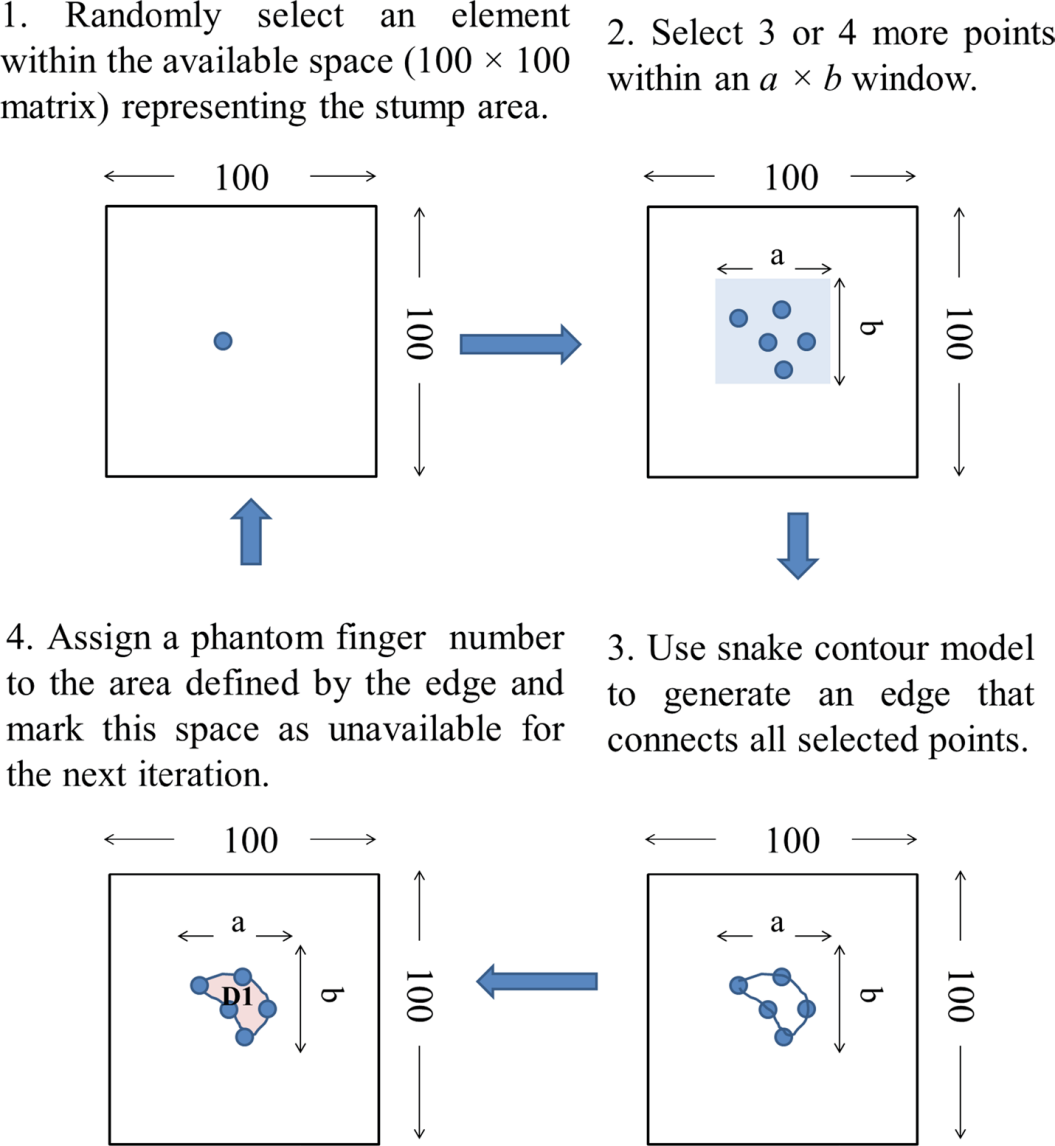
Phantom map generation flow. Phantom map generation flow graph

To simplify the model, it is assumed that there is no overlap between phantom digits. Considering the average size of the remaining stump and the typical minimum two-point discrimination distance, the phantom map was modeled as a 100 $\times$ 100 matrix $\varvec{A}$. Each element in the matrix is assigned a number from [0, 1, 2, 3, 4, 5], representing no phantom sensation, phantom thumb, phantom index, phantom middle, phantom ring, and phantom little finger, respectively. After having selected the actual phantom map types (number of phantom fingers—either 5 or 10—and completeness/incompleteness, a contour model was used to generate the individual phantom maps. The generation algorithm starts by randomly selecting 4–5 points within an $a \times b$ window (for 5 finger phantom maps, $0 < a,b \le 60$ and for 10 finger phantom maps, $0 < a,b \le 45$). The values of *a* and *b* were determined empirically to provide reasonable phantom map shapes and a wide range of phantom sensation coverage; for example, when too large it was very difficult for the contour algorithm to converge and/or generate balanced and representative phantom maps. The selected data points are connected by an active snake contour model [26]. All the elements included in the contour edge are assigned the same finger number, starting from 1. Then the generation algorithm continues selecting data until all the needed phantom fingers are assigned (Fig. 3).

The generated phantom maps are then used to test the performance of the proposed detection algorithms.

The size of the phantom sensation area (the area on the remaining stump that can evoke the sensation of the lost fingers) varies from person to person. Thus, we define a variable called ‘phantom sensation coverage ($C_{\text {PS}}$)’ to describe the ratio of the phantom sensation area against the remaining stump

1 $$\begin{aligned} C_{\text {PS}} = \frac{ A_{\text { Phantom fingers}}}{A_{\text { Stump area}}}, \end{aligned}$$

where $A_{\text { Phantom fingers}}$ is the total phantom finger area, and $A_{\text { Stump area}}$ is the whole stump area, $A_{\text { Stump area}} = 100 \times 100$.

**Fig. 4:**
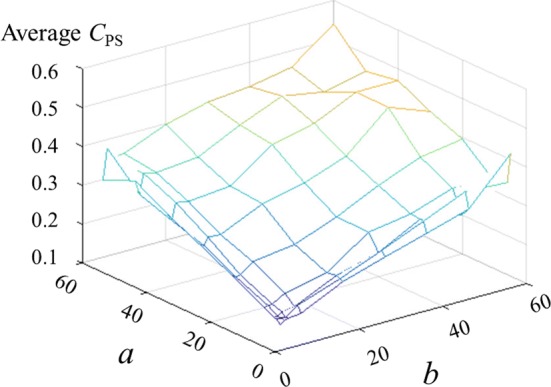
CPS controlled by a and b. Phantom sensation coverage control: average $C_{\text {PS}}$ of 5 finger phantom maps generated by varying *a* and *b* within $0<a,b \le 60$

**Fig. 5:**
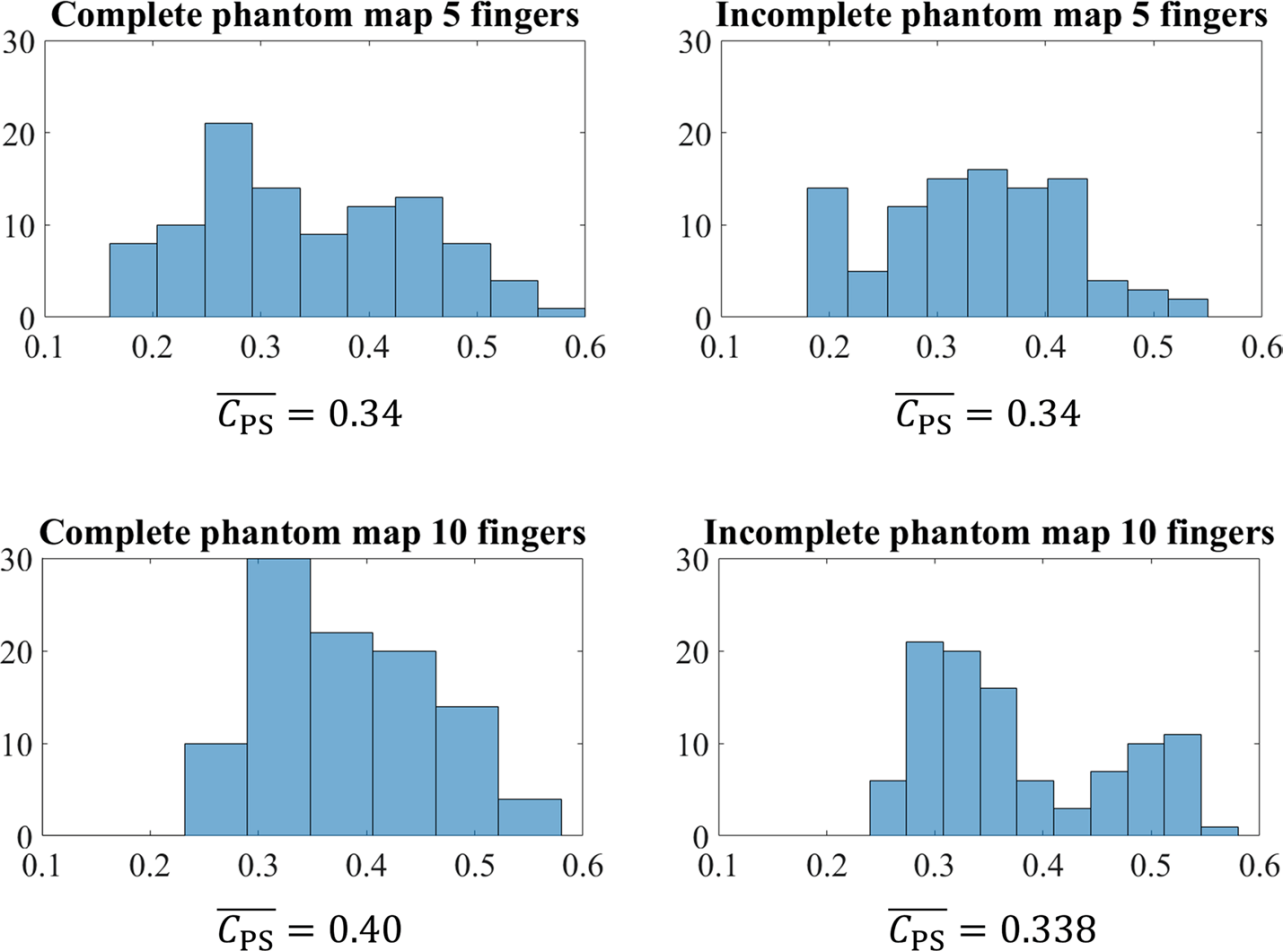
CPS distribution. $C_{\text {PS}}$ distribution of 400 generated phantom maps (100 samples of each type). x-axis: $C_{\text {PS}}$, y-axis: number of phantom maps.

**Fig. 6:**
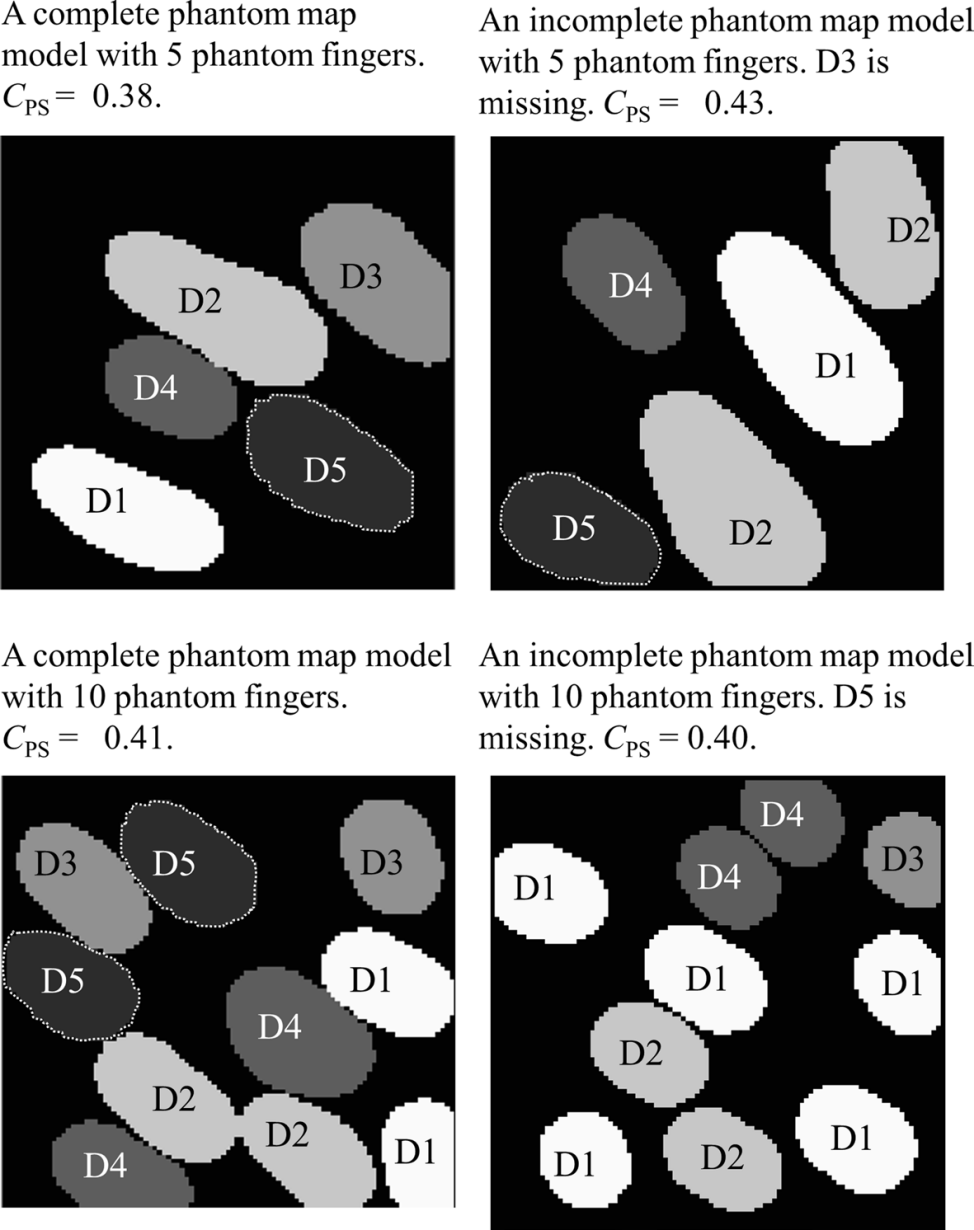
Generated phantom map models. Examples of generated phantom map models

The phantom map model generation method provide the possibility to adjust the phantom sensation coverage range (Fig. 4), select between complete and partial phantom maps, and control the total number of generated phantom digit representations (Fig. 5). Examples of generated phantom map models are shown in Fig. 6.

### Database from reported phantom map images

To further validate the proposed algorithms, we also used five reported phantom map images from the literature [13, 23, 27, 28] to build a second phantom map database. To digitize the reported phantom map images, the edge of each phantom finger in the reported phantom map images (Fig. 1a) was outlined in Illustrator (Adobe Illustrator CC, United States) and each phantom finger area is assigned a color. Then each Illustrator processed image is imported into MATLAB 2017b (The MathWorks, Inc., United States) and down-sampled into a $100 \times 100$ matrix (Fig. 1b), with each color mapped to its corresponding grey scale value. The compressed matrix (image) is used for classification. The corresponding predicted phantom maps using OVO-SVM and $2 \times 2$ majority pooling are presented in Fig. 1c.

**Fig. 7:**
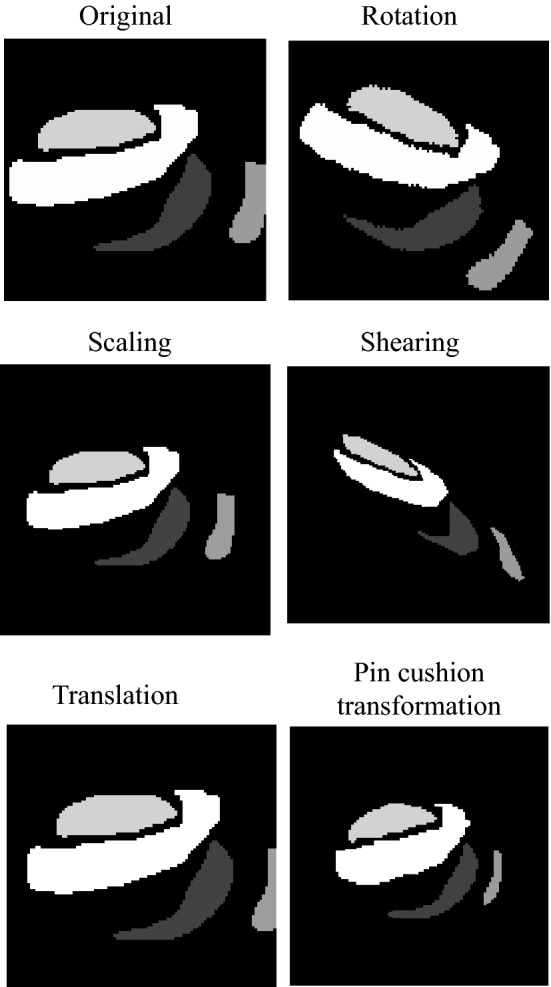
Examples of reported phantom map image transformations, including rotation, scaling, shearing, translation, and pin cushion transformation in contrast to the original phantom map shape

The digitized phantom maps are then transformed into a group of images using rotation, scaling, shearing, translation, and barrel or pin cushion transformation. For rotation, each digitized reported phantom map image is rotated between 0${^\circ }$ and 360${^\circ }$ for every 5${^\circ }$. For scaling, both proportional scaling and one-dimensional scaling are used. The scaling factor ranges from 10 and 100%. For the translation, both single direction and bi-directional translation are used. The shear factor ranges from 0 to 1. For barrel or pin cushion transformation, the amplitude of the cubic term varies between − 0.01 to 0.01. Examples of the transformed images are shown in Fig. 7.

## Sampling methods

In our current work, we apply machine learning algorithms to accurately detect phantom map shapes with limited number of sampled points, referred as samples in the rest of the paper. For machine learning, selecting representative training data is essential, especially in applications where labeling is expensive. From our experience working with amputees, it can be observed that (a) the amputee can give a clear response to the location of the stimuli (i.e., clearly identify which phantom finger has been touched), and (b) when the stimulation falls across several phantom fingers, the amputee can still indicate which finger felt stronger.

Taking these elements into account, three different sampling methods for SVMs are proposed and explained in this section (Fig. 2: Step 1: Sampling): random sampling, systematic sampling, and majority pooling sampling. Although their effectiveness is explored below on simulated data, the sampling protocols are designed in such a way that they are also to be applicable in future clinical tests.

### Random sampling (RS)

**Fig. 8:**
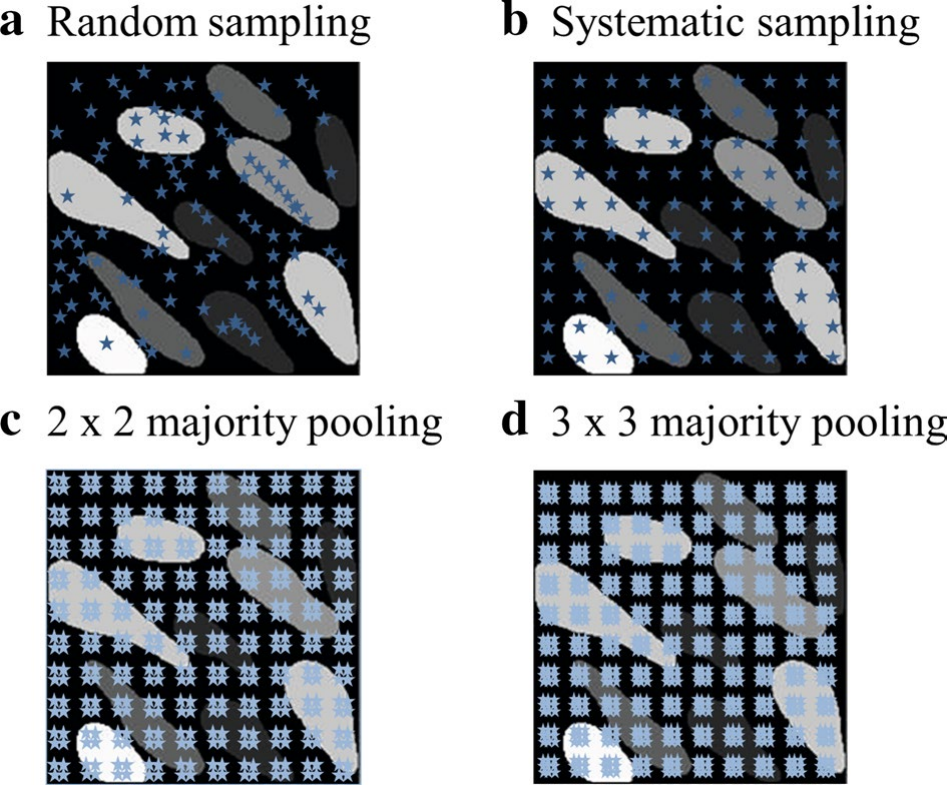
Illustration of the proposed sampling methods: **a** random sampling, **b** systematic sampling, **c** 2 × 2 majority pooling sampling, and **d** 2 × 2 majority pooling sampling. The total number of samples is 100 for each case. The stars represent sampled points. The sampled points were enlarged for better visualization

Random sampling consists of randomly picking *m* data sets and labeling them individually (Fig. 8a). The *m* data sets will be used for training the support vector machine algorithms.

### Systematic sampling (SS)

Instead of randomly choosing the query data point, the whole phantom map region is evenly divided into a regular grid. Each grid point is a sampling point (Fig. 8b).

### Majority pooling sampling (MPS)

The idea of majority-pooling comes from the max-pooling concept of convolutional neural networks. Applying pooling can result in more compact representations and higher robustness to noise [29]. First, the algorithm defines a set of non-overlapping rectangular windows $\varvec{W_{i}}$ each containing $p \times q$ sampling points, where $\varvec{W_{i}} \subset \varvec{A_{\text {stump area}}}$ and $i \in [1, 2 \ldots , M]$, *M* being the number of training data sets. Then the corresponding phantom finger numbers of all the data points within $\varvec{W_{i}}$ are collected. In clinical practice the subject responds which finger(s) he or she feels being touched, and if more than one phantom finger is within the stimulation region, the amputee has to choose which one feels stronger. In this study, all the points within the selected window will be labeled as the one the amputee chooses, i.e.,

2 $$\begin{aligned} \forall i, \varvec{W}_{i} = \text {Mo}(\varvec{W}_{i}), \end{aligned}$$

where $\text {Mo}$ represents mode operation, which selects the value which occurs most frequently in the window.

Examples of majority pooling sampling are shown in Fig. 8c, d. Majority-pooling can introduce errors because all the data points within a pooling window are labeled according to the majority vote. A pooling induced error $E_{\text {MP}}$ is therefore defined as

3 $$\begin{aligned} E_{\text {MP}} = \frac{N_E}{N}, \end{aligned}$$

where $N_E$ is the number of wrongly labeled training data due to pooling and *N* is the total number of training data sets.

## Support vector machine

After selecting the training samples, the support vector machines (SVMs) need to be trained using the acquired samples (Fig. 2: Step 2: training). After the training step, the SVMs can be used to classify the phantom map shape distribution (Fig. 2: Step 3: Classification).

### Support vector machine basics

A SVM is a non-probabilistic, parametric binary linear classifier, based on the maximum margin principle. The basic idea behind a SVM is to minimize the classification error rate while maximizing the geometric margin between two classes [30].

In a binary-class classification problem, given *M* training data sets: $$ \mathbf {T_r} =\{ (\mathbf {p_{tr,1}}, c_1), (\mathbf {p_{tr,2}}, c_2), \ldots , ( \mathbf {p_{tr,M}}, c_M) \} $$, where $$\mathbf {p_{tr,i }}\in \mathbf {R^n} $$, $$i =1,2, \ldots ,M$$, $$\mathbf {p_{tr,i}}$$ is the feature of the *i*th training data set, $$\mathbf {R^n}$$ is the feature space, *n* is the feature dimension, and $$c_i $$ is the training class label in the *i*th training data set, whereby $$c_i \in \{-1, +1\}$$, $$i =1,2, \ldots ,M$$ (outputs of Fig. 2: Step 1). The SVM training consists in solving the following optimization problem:

4 $$\begin{aligned} \begin{array}{ll} \text {min} \quad \frac{1}{2} \begin{Vmatrix} \varvec{ \omega } \end{Vmatrix} ^2 + C \sum _{i=1}^{M} \zeta _{i}, \\ \text {s.t.} \quad c_i(\varvec{\omega } ^T\varvec{ p_{\text {tr}}} + b) \ge 1 -\zeta _i, \\ \text {and} \quad \zeta _i \ge 0, \quad  i = 1 \ldots M, \end{array} \end{aligned}$$

where *b* is the bias of the hyperplane, *C* is the penalty parameter, and $\zeta _i$ is the slack variable.

Because the relationship between class labels (phantom digit number) and attributes (the location of sampling points) is non-linear, a non-linear kernel function is used to map the input space into a feature space for higher classification accuracy. In this paper, a radial basis function (RBF) kernel is chosen [31], because it maps the input space into Hilbert space (an infinite hyperplane) and provides more flexibility [32]. More details about the kernel function and SVM parameter tuning can be found in Appendix A.

### Training and classification data definition for automatic hand phantom map detection

As mentioned before, SVMs use collected training data sets, including training features and training classes to find support vectors (a selected number of training data). Then SVMs use the support vectors to assign each testing feature a class. The training data sets collection $\mathbf {Tr}$ is defined as

5 $${\mathbf{Tr}} = \{({\mathbf{p}}_{\mathbf{{tr,1}}}, c_{tr,1}), \ldots ,({\mathbf{p}}_{{\mathbf{tr,M}}}, c_{tr,M}) \},$$

where $M = 100$ when using random and systematic sampling, and $M = 100 \times p \times q$ when using majority pooling sampling ($p \times q$ being the pooling size), $\mathbf {p_{tr,i}}$ is the training feature (the position of the data points in a phantom map), with $\mathbf {p_{tr,i}} = (x_i, y_i)$, $x_i$ and $y_i$ being the coordinates of point *i* in a phantom map matrix *M*, and $c_i$ is the class label ($c_i \in \{0, 1, 2, 3, 4, 5\}$, $i = \{1, 2, \ldots , M\}$).

The collection of testing features is defined as

6 $$\begin{aligned} \mathbf {P_{tst}} =\{ \mathbf {p_{tst,1}}, \ldots ,\mathbf {p_{tst,10000}} \}, \end{aligned}$$

whereas the collection of testing classes is defined as

7 $$\begin{aligned} \mathbf {C_{\text {tst}} }=\{ c_{\text {tst},1}, \ldots ,c_{\text {tst},10,000} \}, \end{aligned}$$

where $c_{\text {tst},1} \in {[ 0, 1, 2, 3, 4, 5]}$. For each individual phantom map, *M* selected points are sampled and used to train the SVMs. Because the distribution of phantom map is different from person to person, each individual phantom map needs its own training and classification.

### Multi-class support vector machine

A SVM is intrinsically a binary classifier. Two approaches have been proposed to extend binary SVM classifiers to multi-class SVM classifiers. The first approach, proposed by Crammer and Singer, is a direct method, which treats the multi-class classification problem as a large ‘constrained optimization problem with a quadratic objective function’ [33]. The second approach is to decompose a multi-class classification problem into a collection of binary classification problems. The direct approach is slow and involves a complex optimization problem. The decomposition approaches generally offer good performance and are easier to implement [34].

There are mainly four decomposition methods: one-vs-all (OVA), one-vs-one (OVO), the directed acyclic graph (DAG), and the binary tree (BT) method.

#### One-vs-all support vector machine (OVA-SVM)

The principle of an OVA-SVM is to train each class against all the rest of the classes. When training the *j*th class, the *j*th class is assigned positive labels, while the others are assigned negative labels. After training all the binary classifiers, the final class is determined by the highest output value.

#### One-vs-one support vector machine (OVO-SVM)

For an OVO-SVM, all the combinations of class pairs need to be trained. For a *k*-class classification problem, each binary classifier determines a preferred class. After training all the $k(k-1)/2$ classifiers, the class that has the most votes wins. Although an OVO-SVM needs to train more binary SVMs, the training data contained in each subset is smaller. Thus, the training time needed for each individual SVM is smaller, compared to that of an OVA-SVM.

#### Directed acyclic graph support vector machine (DAG-SVM)

The training of a DAG-SVM is the same as OVO-SVM. In the classification phase, it follows a rooted binary directed acyclic graph (DAG). For a *k*-class classification problem, this graph has $k(k-1)/2$ internal nodes and *k* leaves. Each node is a binary classifier $D_{i,j}$. Each leaf represents a class. *k* is the total number of classes [35]. In our application, the DAG would be [0 1 2 3 4 5] for a complete phantom map, where 0 represents no phantom sensation and 1–5 correspond to the five phantom fingers. Then each binary SVM chooses the preferred class between the first and the last class of the list. The non-preferred class is then deleted from the list. This procedure continues until a final class decision is reached.

#### Binary tree support vector machine (BT-SVM)

A BT-SVM is constructed based on a binary tree structure. Each internal node is a binary SVM. During the training phase, half of the remaining training data are assigned positive labels, the other half negative labels. The main goal of a BT-SVM is to reduce the number of binary classifiers, thus decreasing the needed training and classification time.

Table 1 lists the number of binary SVMs required for a *k*-class classification problem.

**Table 1:** Multi-class SVM classifiers: number of binary SVMs required for the four main decomposition methods

Method	Number of binary SVMs required for a k-class classification problem	Number of SVMs required to classify a complete phantom map
OVA	*k*	6
OVO	$\frac{k(k-1)}{2}$	15
DAG	$\frac{k(k-1)}{2}$	15
BT	2$log_2k$	5

### Fuzzy support vector machines

Although the classification accuracy of a SVM is very high, it is highly influenced by noise and outliers [36]. Fuzzy SVM (FSVM) was proposed to increase the robustness of a conventional SVM by applying a fuzzy membership function to the training data sets [37]. The fuzzy membership function is used to reformulate the SVM so that noisy input points contribute less in training the decision surface. In our classification application and within the proposed model, the noise mainly comes from majority pooling sampling (“Majority pooling sampling (MPS)” section). In order to reduce the effect of pooling-induced errors, a step fuzzy membership function $f_c$ is therefore proposed:

8 $$\begin{aligned} f_c = \left\{ \begin{array}{ll} 1 \; \; {\text {when}}\quad \forall \,i \, and \, j, \, i \ne \, j, \, S_i\, =\, S_j, \\ \alpha \; \; \text {otherwise}, \\ \end{array} \right. \end{aligned}$$

where *i* and *j* are the element indices in a pooling window, $S_i$ and $S_j$ are the phantom sensation labels of the *i*th and *j*th element, with $S_i , S_j\in [0, 1, 2, 3, 4, 5]$, and $\alpha$ is a constant.

In this approach, the penalty parameter *C* in (4) becomes an array:

9 $$\begin{aligned} \mathbf {C} = C_{\text {const}} \times f_c, \end{aligned}$$

where $C_{\text {const}}$ is the penalty parameter value in a conventional SVM.

### Active learning support vector machine

Based on the consideration that the human phantom map detection process is likely gradual and adaptive, we decided to apply active learning as well to the four decomposition SVMs. Active learning is able to query the candidate data interactively, using a specified rule (called query strategy) and sequentially adding new data for labeling and contribution to the training [38]. One of the most widely used query strategies is uncertainty sampling [39, 40], whereby margin sampling and its variations, especially multi-class level uncertainty, have shown good performance when combined with SVMs [39]. Moreover, in order to achieve faster training, batch-mode active learning is often used, whereby a group of instances is added at a time. Furthermore, diversity criteria are often employed for query data selection to ensure the representativeness of selected instances. In the current study, we applied angle-based diversity SVMs [41, 42].

Different initial and batch sizes with and without diversity criteria were employed in this study. During preliminary testing and parameter tuning, we found out that (a) the diversity criteria did not improve the classification accuracy, and (b) the initial size 80 with batch size 2 achieved the highest classification accuracy. In the following sections, only results using the aforementioned testing configuration will be presented.

## Results

In the absence of a wearable dense stimulation array, the aforementioned algorithms were tested on the two databases detailed in “Hand phantom map model generation” section, containing 400 generated phantom map models (Fig. 5), as well as processed and transformed reported phantom map images, respectively. Both the phantom map models and the phantom map detection methods were implemented in MATLAB 2017b (The MathWorks, Inc., United States). The program was running on a HP laptop with an Intel core i5-4300 CPU@1.90GHz.

We have opted for a total of 100 samples for the sampling phase based on initial testing results, which highlighted that a sampling size smaller than 100 led to a large increase in error rate, while the accuracy increase was not significant above this size. We also believe this number to be realistic for a dense array. Indeed, from our experience of testing with amputees, the average time needed to stimulate and get the response is 15–30 s. In practice, when the total number of samples is 100, 25–30 min are needed to complete data collection (the active time involving an amputee). This time is deemed acceptable without causing fatigue nor adaptation.

The overall simulation setup is summarized in Table 2. The accuracy (“Accuracy” section) and timing (“Timing” section) results are presented and discussed below.

**Table 2:** Simulation setup for the different sampling methods

	Sampling methods	
	RS and SS	MPS (pooling size = $p \times q$)
# Training data sets per phantom map model	100	$100 \times p \times q$
# Testing data sets per phantom map model	10,000	
Number of phantom maps in each database		
Phantom map database	Type	Number of phantom map images
# Generated phantom maps	Complete 5	100
	Complete 10	100
	Incomplete 5	100
	Incomplete 10	100
# Transformed reported phantom map images	Reported (original)	5
	Rotation	72 $\times$ 5
	Scaling	10 $\times$ 5
	Shearing	20 $\times$ 5
	Translation	10 $\times$ 5
	Barrel or pin cushion	20 $\times$ 5

### Accuracy

The accuracy are presented by six types of metrics, defined in “Accuracy metrics definition” section. The influences of two types of stimulation arrays: dense and coarse, SVM parameters, sampling methods, different SVM algorithms, and socket shifting on accuracy are presented.

#### Accuracy metrics definition

To evaluate the accuracy of the phantom map detection algorithms, six types of metrics are defined: absolute error rate ($E_A$), functional error rate ($E_F$), redundancy error rate ($E_R$), insufficiency error rate ($E_I$), precision error rate ($E_P$), and phantom sensation coverage ratio ($R_\text {PSC}$) between a generated phantom map and its corresponding predicted phantom map.

The general error rate *E* is defined as

10 $$\begin{aligned} E = \frac{ \sum _{i=1}^{N} f_i(c_{i,a},c_{i,p}) }{N}, \end{aligned}$$

$$\begin{aligned} \text {where} \; f_i(c_{i,a},c_{i,p}) = \left\{ \begin{array}{ll} 1 \quad \text {when} \; c_{i,a} \ne c_{i,p},\\ 0 \quad \text {when} \; c_{i,a} = c_{i,p},\\ \end{array} \right. \end{aligned}$$

$$ c_{i,a}$$ is the real label of the $$i{\text {th}}$$ testing set, $$ c_{i,p}$$ is the predicted label of the $$i{\text {th}}$$ testing set, and *N* is the number of testing data sets for $$E_A$$, $$E_F$$, $$E_R$$, and $$E_I$$, and the number of testing data sets containing phantom sensation for $$E_P$$.

- For $E_A$, $c_{i,a} \in \{0, 1, 2, 3, 4, 5 \}$, $c_{i,p} \in \{0, 1, 2, 3, 4, 5 \}$. $E_A$ measures the fraction of all misclassified data points of a predicted phantom map.
- For $E_F$, $c_{i,a} \in \{1, 2, 3, 4, 5 \}$, $c_{i,p} \in \{1, 2, 3, 4, 5 \}$. $E_F$ measures the fraction of points belonging to one phantom finger which are falsely classified into another finger, leading to a functional error (wrong finger stimulation when providing sensory feedback). ($E_F$ = 1-*recall* [43]).
- For $E_R$, $c_{i,a} = 0$, $c_{i,p} \in \{1, 2, 3, 4, 5 \}$. $E_R$ measures the fraction of points belonging to class 0 (i.e. no phantom sensation) which are wrongly classified into other classes. When providing sensory feedback, these points do not cause mistakes between fingers, but their stimulation is redundant and costs energy without providing useful feedback.
- For $$E_I$$, $$c_{i,a} \in \{1, 2, 3, 4, 5 \}$$, $$c_{i,p} = 0 $$. $$E_I$$ measures the loss of stimulation points which takes place when data points belonging to class 1 to 5 (phantom thumb to phantom little finger) are misclassified as class 0 (no phantom sensation) and therefore not stimulated.
- For $E_P$, $c_{i,a} \in \{1, 2, 3, 4, 5 \}$, $c_{i,p} \in \{1, 2, 3, 4, 5 \}$. $E_P$ is an extension of the *precision* measurement of binary classification [43]; it indicates the fraction of incorrectly classified phantom sensation points with respect to all the phantom sensation points in the generated phantom map.

The relationship of the first four types of error rates is:

11 $$\begin{aligned} E_A = E_F + E_R + E_I. \end{aligned}$$

$E_P$ is related to $E_F$:

12 $$\begin{aligned} E_P =\frac{ E_F }{ C_{\text {PS}}}. \end{aligned}$$

$R_{\text {PSC}}$ is defined as

13 $$\begin{aligned} R_{\text {PSC}} = \frac{C_{\text {PS}}'}{C_{\text {PS}} }, \end{aligned}$$

where $C_{\text {PS}}'$ is the phantom sensation coverage of the predicted phantom map and $C_{\text {PS}}$ is the phantom sensation coverage of the original generated phantom map. $R_{\text {PSC}}$ defines the proportion of the predicted phantom map $C_{\text {PS}}'$ over the corresponding generated phantom map model (the original $C_{\text {PS}}$) (13).

**Fig. 9:**
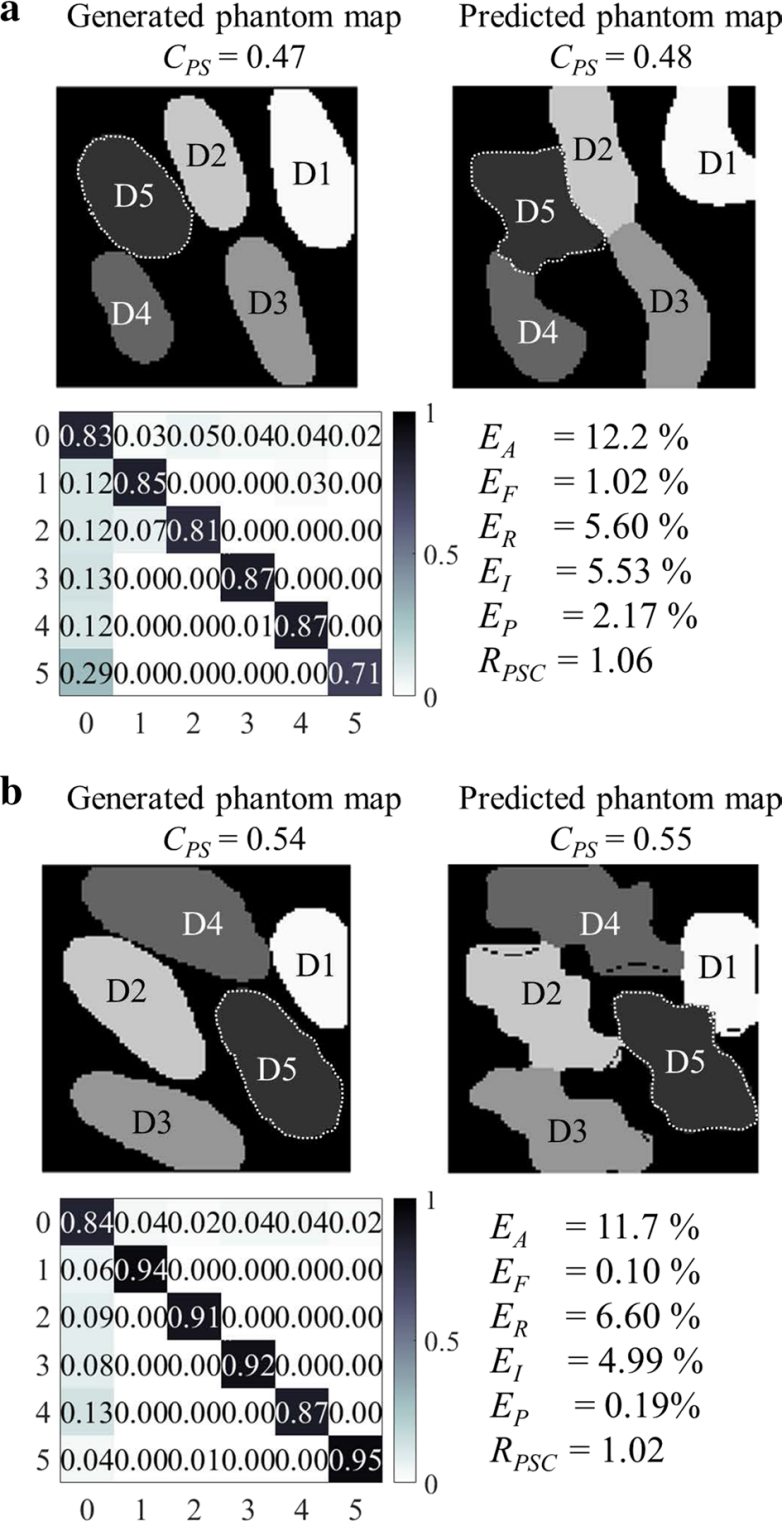
Examples of real and predicted phantom maps. Examples of generated phantom maps, predicted phantom maps, their confusion matrices, absolute error rates ($E_A$), functional error rates ($E_F$), redundancy error rates ($E_R$), insufficiency error rates ($E_I$), and precision error rates($E_P$) using **a** BT-SVM with $3 \times 3$ majority pooling and **b** OVA-SVM with $3 \times 3$ majority pooling

To demonstrate the defined metrics, Fig. 9 shows examples of generated phantom maps, predicted phantom maps, their confusion matrices and accuracy metrics.

#### SVM parameters *C* and $\gamma$

**Fig. 10:**
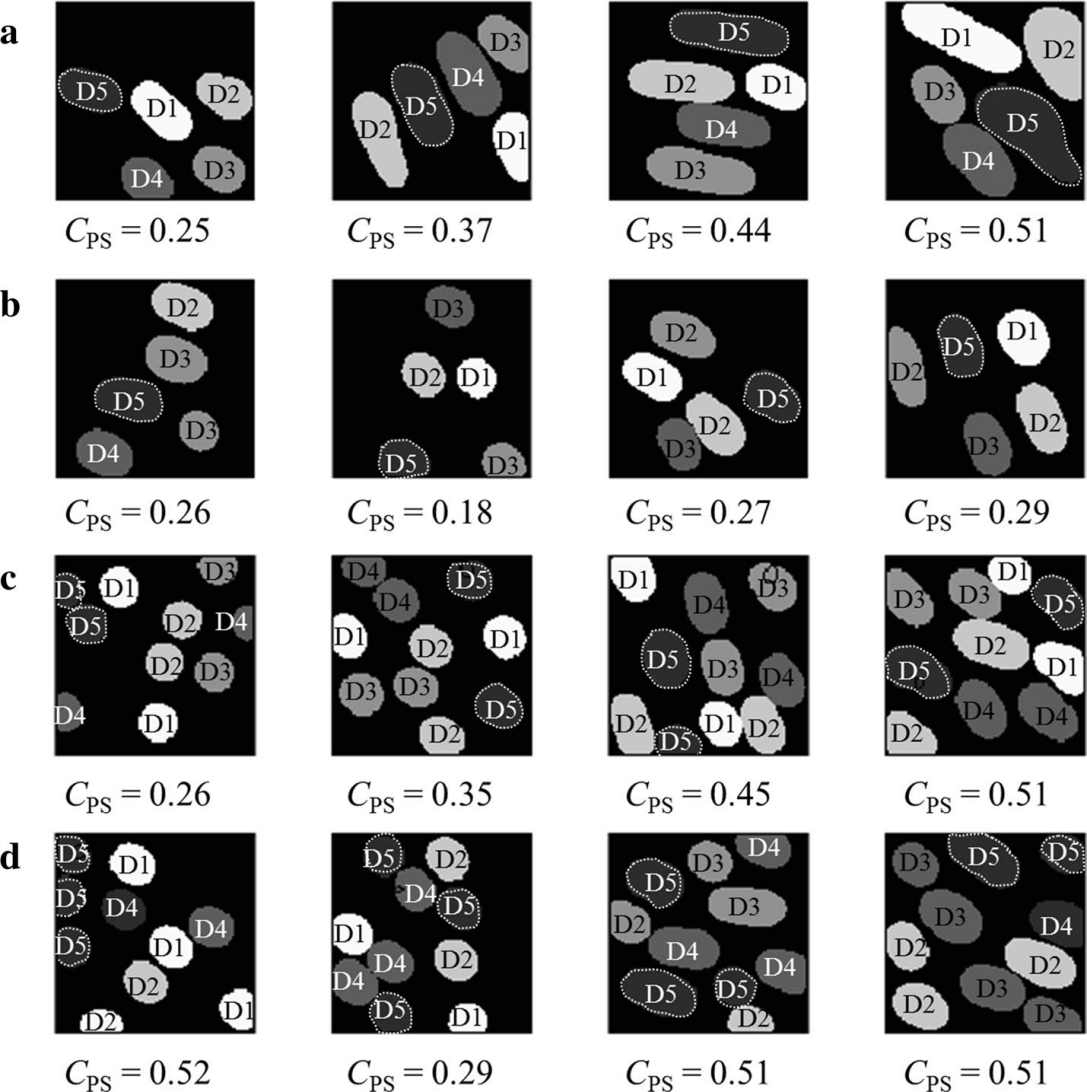
Selected phantom maps PSC. The 16 pre-selected representative phantom maps used for choosing the SVM parameters *C* and $\gamma$. **a** Four complete phantom maps with 5 phantom fingers, **b** four incomplete phantom maps with 10 phantom fingers, **c** four complete phantom maps with 10 fingers, and **d** four incomplete phantom maps with 10 fingers

For SVM-based methods, the parameter selection exerts a great influence on the classification performance. The involved parameters are the penalty parameter *C* and the RBF kernel parameter $\gamma$. In order to determine the optimal *C* and $\gamma$ values, 16 pre-selected representative phantom maps were used (shown in Fig. 10), carrying out a grid search to cover a range of *C* and $\gamma$ values ($C \in [10^{-3}, 10^{-2}, 10^{-1}, 1, 5, 10, 20, 30, 40, 50]$ and $\gamma \in [10^{-3}, 10^{-2}, 10^{-1}, 1, 5, 10, 20, 30, 40, 50]$). The *C* and $\gamma$ pairs that produce the smallest absolute error rate $E_A$ are selected for further evaluation.

#### Grand average accuracy

**Fig. 11:**
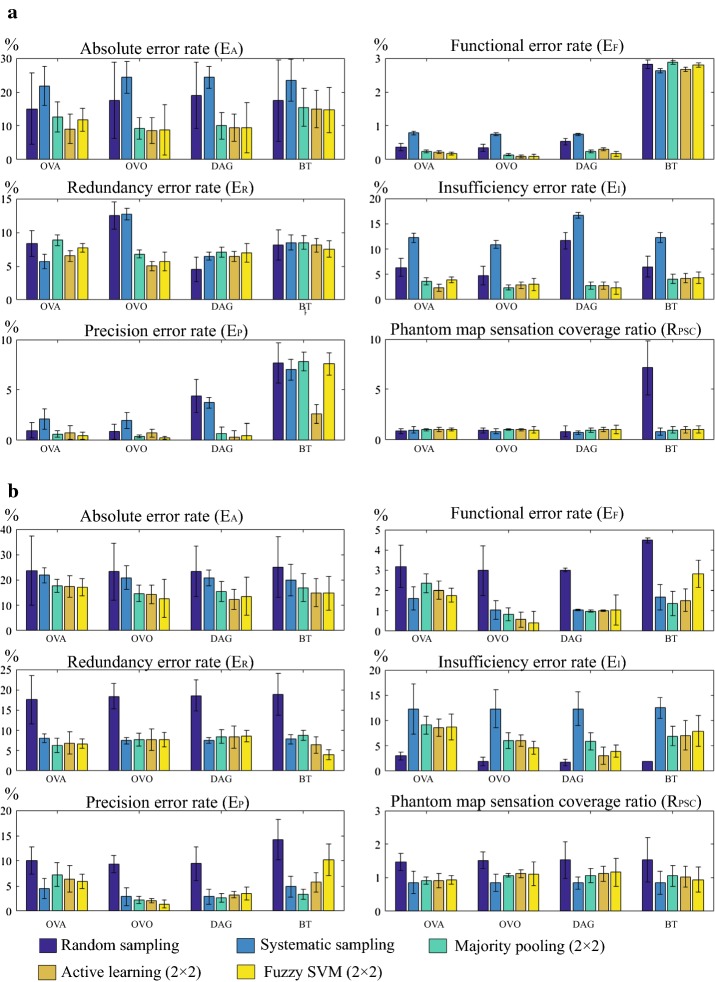
Grand average accuracy using dense array. Grand average error rates and phantom sensation coverage ratios over **a** all 400 generated phantom maps and **b** five reported phantom map images and their corresponding transformed images. For $2 \times 2$ majority pooling, $\overline{E_{\text {MP}}}$ = 5.35% for generated phantom maps and $\overline{E_{\text {MP}}}$ = 4.27% for reported phantom map images. The grand average accuracy is influenced both by the sampling methods and SVM algorithms used. For both generated and reported phantom maps, OVO-SVM produces the smallest error rate. Even though the absolute error rate ($E_A$) for reported phantom maps is higher than for the generated ones, the more critical metric (function error rate $E_F$) is still within an acceptable range

The grand average accuracy is the average error rate over all the phantom maps in one database. The grand average accuracy and phantom sensation coverage ratio of generated phantom maps and reported, as well as their transformed phantom map images are presented separately in Fig. 11.

The accuracy results obtained by using the reported phantom map images showed similar trends as those obtained by using the generated phantom maps. For example, both types of phantom maps benefited from majority-pooling sampling, OVO decomposition architecture, and added fuzzy logic or active learning to the penalty parameters. The following analyses (“Discussion” section) are therefore applicable to both types, although we will focus mainly on the discussion of the generated data. Quantitatively speaking, the error rates of the reported phantom maps are slightly higher than those of the generated ones, for all the algorithms and sampling methods tested. This could be explained by the lower average phantom sensation coverage of the former.

#### Phantom map detection accuracy using coarse arrays

**Fig. 12:**
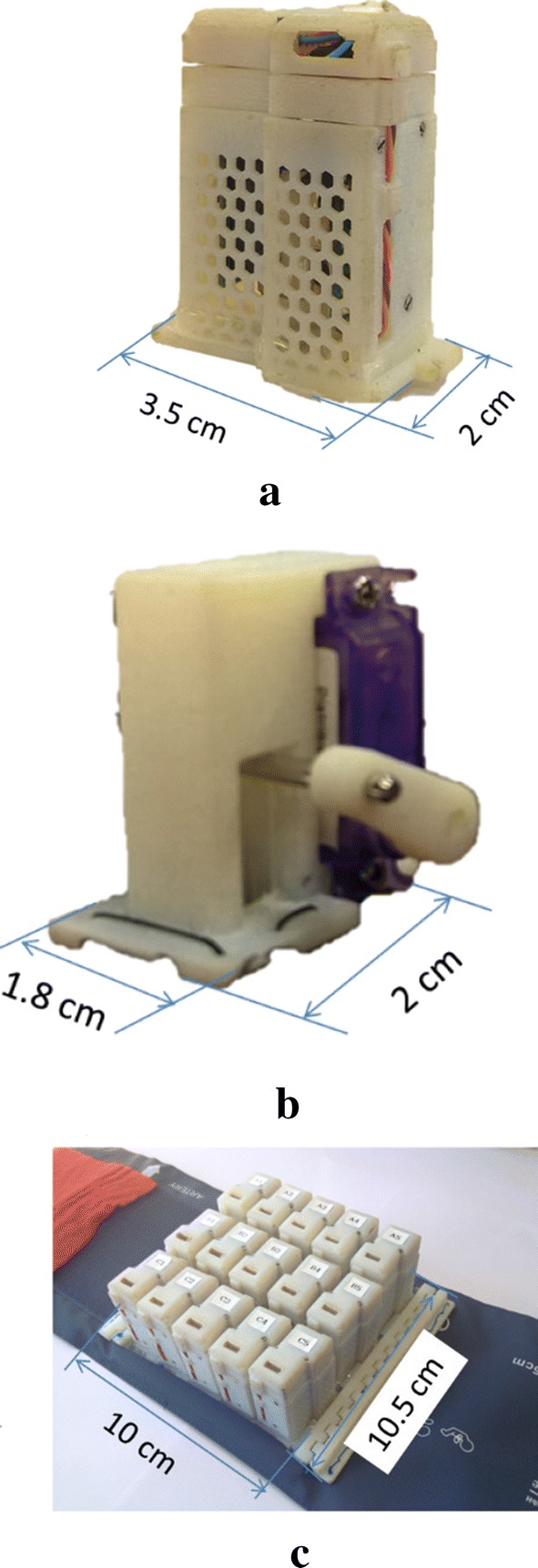
Stimulation devices. Two types of stimulation devices and an experimental coarse stimulation array. These are primarily used for providing sensory feedback for upper limb amputees. **a** Hybrid (vibrotactile and mechanotactile) stimulation device, **b** mechanotactile stimulation device

The potential performance using coarse stimulation arrays designed in our lab is also evaluated. These stimulation arrays are designed primarily to provide sensory feedback for upper limb amputees. Figure 12a is a hybrid stimulation actuator combining a servo motor and an eccentric rotating mass vibrator. The minimal contact size is fixed by the vibrator (153 mm$^2$). Fig. 12b is a servo motor based mechanotactile vibrator; the arm and pin are 3D printed and the contact size is controllable. A $3 \times 5$ hybrid actuator array (Fig. 12c) [44] or a $4 \times 6$ mechanotactile actuator array can fit on the remaining stump of an amputee and are currently being investigated.

**Fig. 13:**
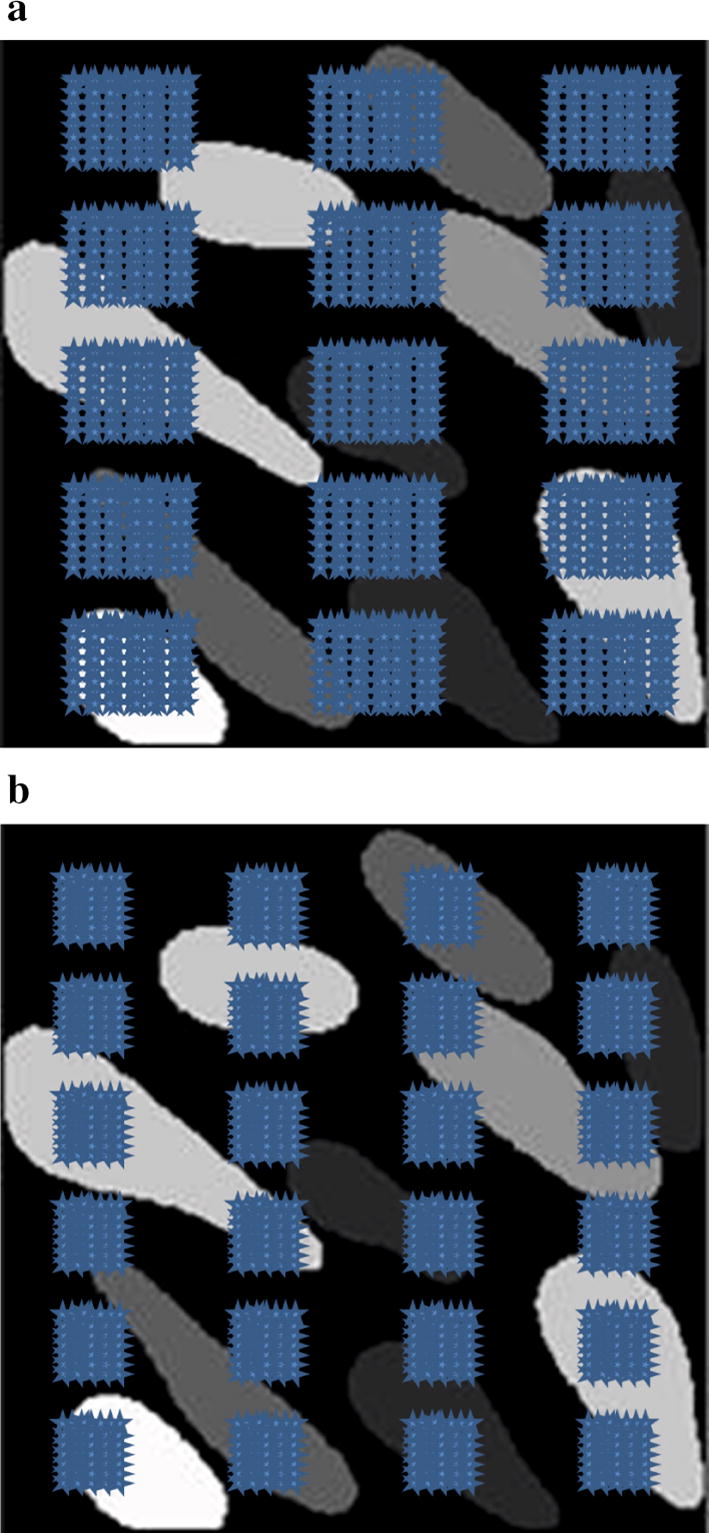
Coarse array sampling methods. Graphical representations of coarse array sampling. The blue stars represent sampling points. **a** Hybrid stimulation array: $3 \times 5$ sampling size with $15 \times 9$ pooling size. **b** Mechanotactile stimulation array: $4 \times 6$ sampling size with $7 \times 7$ pooling size

The average phantom map area is roughly 100 cm$^2$. The minimum actuator contact sizes for mechanotactile and hybrid are 100 and 153 $mm{^2}$, respectively. Given that in a simulation scenario the pooling size reflects the physical contact size, the corresponding minimum pooling sizes $p \times q$ (defined in 4.3) are $15 \times 9$ and $7 \times 7$ for hybrid and mechanotactile actuators, respectively (Fig. 13).

**Fig. 14:**
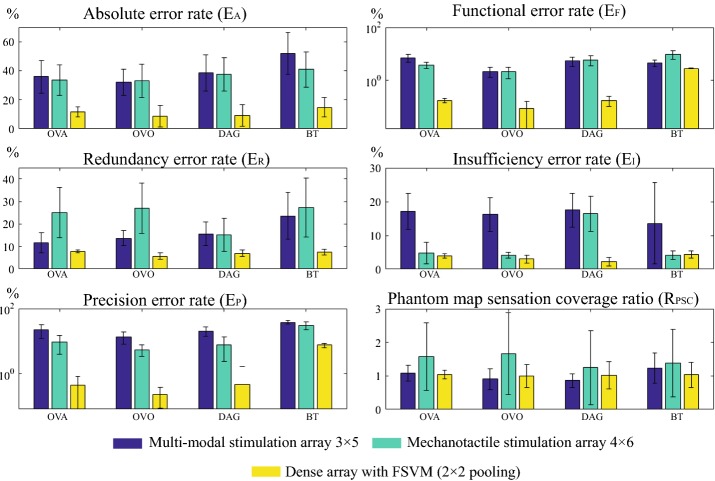
Grand average accuracy using coarse array. Grand average accuracy results over all 400 generated phantom maps using coarse stimulation arrays, compared with the best scenario case in the dense array (Fig. 12). Statistical analysis using paired t-test was conducted. All the coarse array accuracy results were significantly different ($p<0.05$ ) from their counterpart when using the dense array

**Fig. 15:**
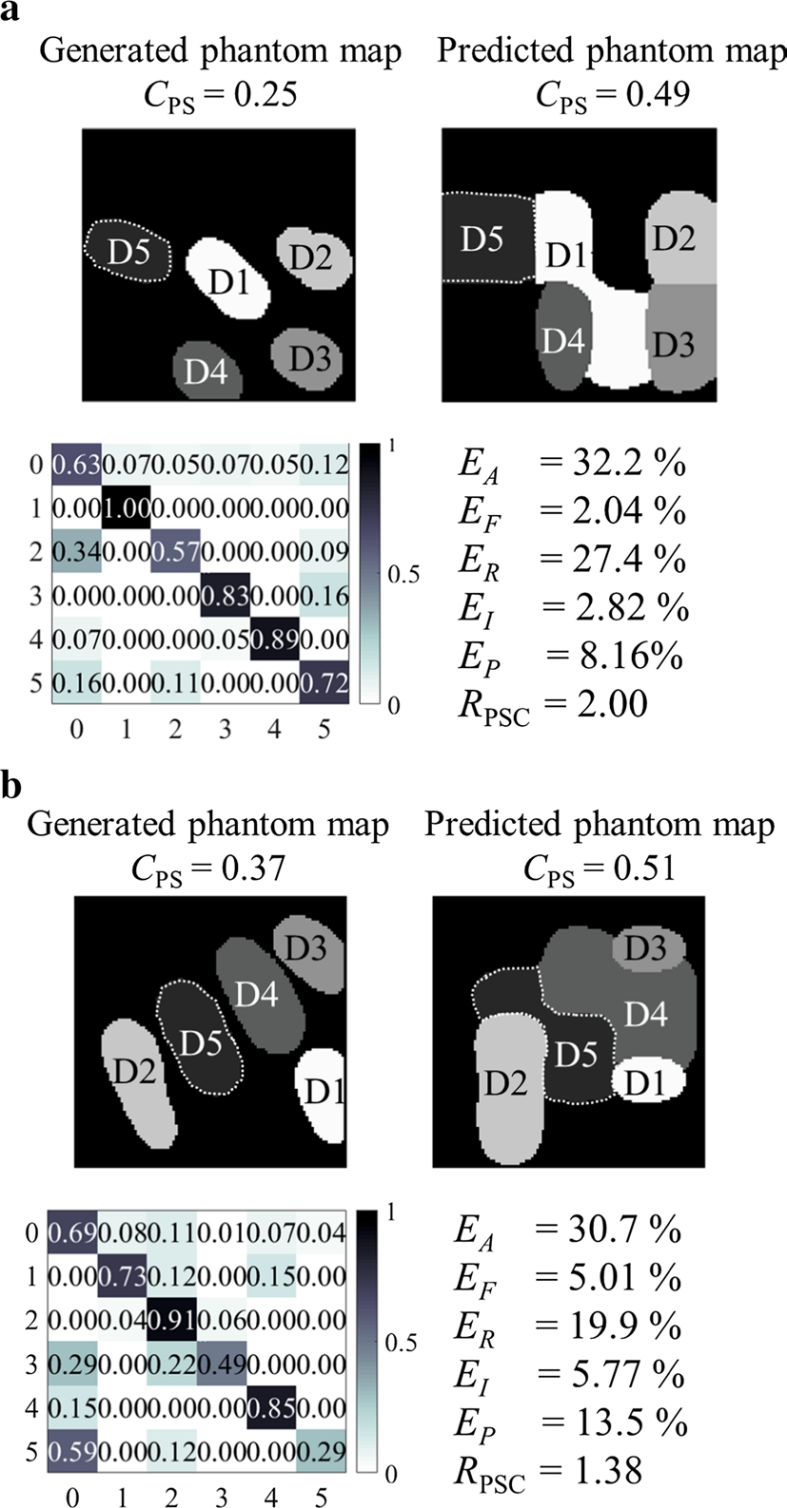
Coarse array examples. Examples of using coarse stimulation arrays to detect phantom map distributions. The used array types and algorithms are **a** OVO-SVM, $3\times 5$ hybrid coarse array (corresponding to $15 \times 9$ majority pooling), and **b** BT-SVM, $4 \times 6$ mechanotactile coarse array (corresponding to $7 \times 7$ majority pooling)

Figure 14 shows the grand average accuracy results of five types of error rates and phantom sensation coverage ratios when using coarse array to detect phantom map shapes, compared with the best case scenario from generated phantom maps and reported and transformed phantom maps. Fuzzy SVMs were also evaluated; the overall performance was however not significantly improved when using FSVMs ($p = 0.34$ when using the paired t-test to compare the results from standard SVMs and fuzzy SVMs), and the corresponding results are therefore not reported. Fig. 15 shows examples of generated phantom maps, their corresponding predicted phantom maps, the confusion matrices, and six metrics.

#### Systematic error caused by socket shifting

**Fig. 16:**
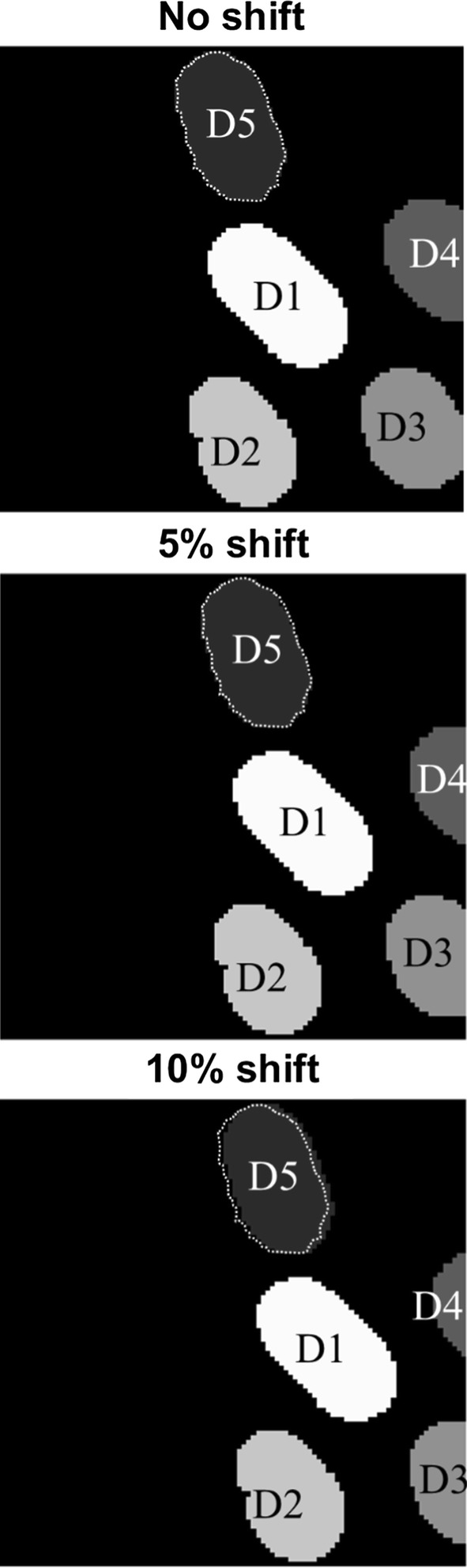
Shift examples. Examples of shifting error caused by a lateral socket shift

**Fig. 17:**
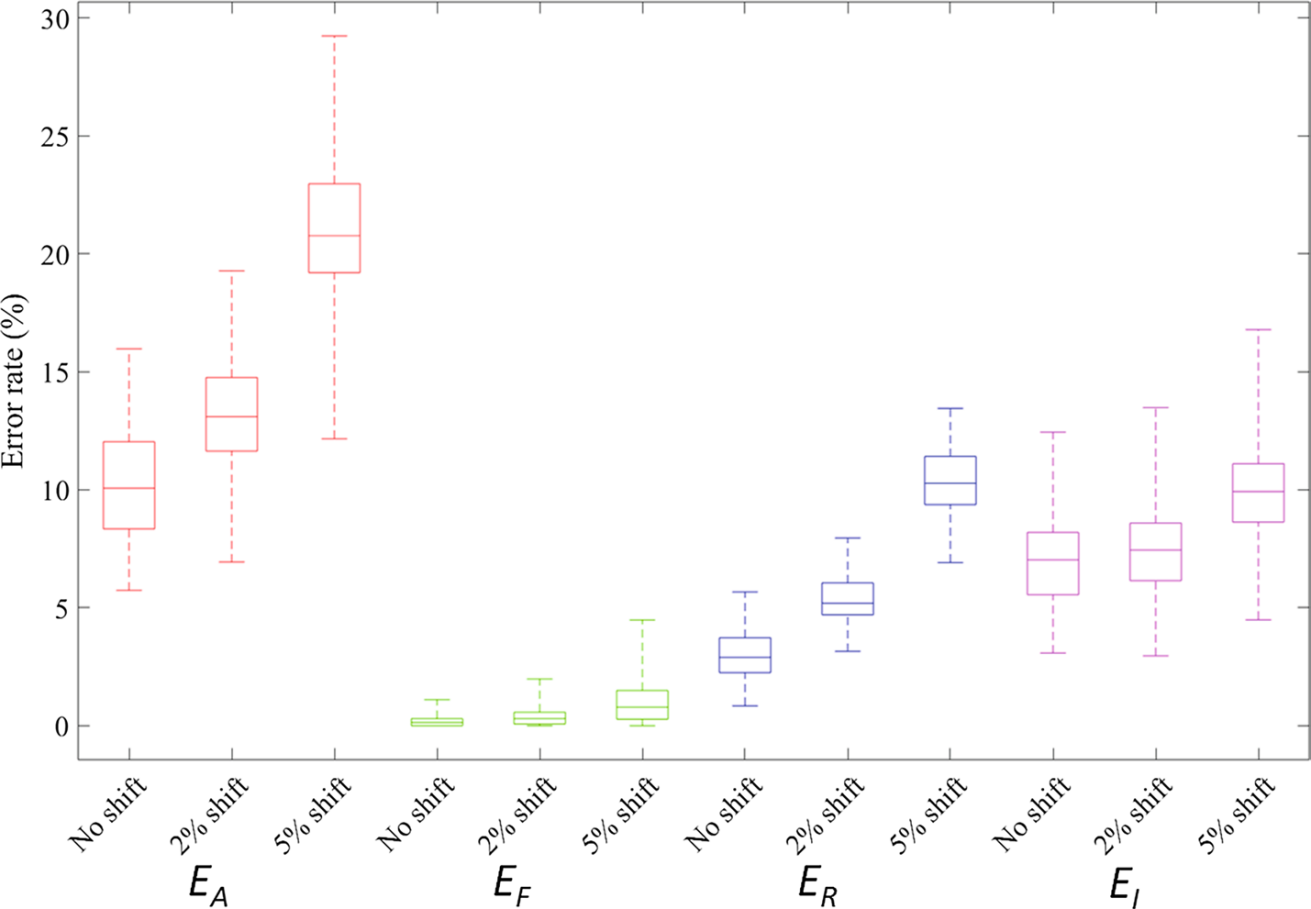
OVO shift error boxplot. Error rates ($E_A$: red, $E_F$: green, $E_R$: blue, and $E_I$: Magenta, $E_P$: black) as functions of different degrees of shifting (no shift, 2% shift, and 5% shift). The rectangle spans the first and the third quartile of the error rate. The line inside each rectangle shows the median value. The two whiskers above and below each rectangle show the minimum and the maximum. The phantom map models used are 100 complete phantom maps with five fingers. The algorithm used was OVO-SVM with $2 \times 2$ majority pooling

After detecting the phantom map shape, the stimulation devices will be used to provide sensory feedback. The stimulation devices themselves are embedded in a socket, which the amputees will need to wear on and off on a daily basis. The socket position can therefore shift, for example laterally as shown in Fig. 16. We have therefore simulated the effect of such a movement on the different error rates ($E_A$, $E_F$, $E_R$, $E_I$, and $E_P$) in case of the OVO-SVM algorithm applied to a scenario using complete phantom maps with 5 fingers each, for different expected shift levels (Fig. 17).

### Timing

Different sampling and training methods result in different training ($T_t$) and classification times ($T_c$). Table 3 shows the grand average training and classification time using different sampling methods, averaged over all 400 generated phantom maps and calculated for the target ideal dense array as well as for the two examples of coarse stimulation arrays currently under investigation to provide sensory feedback (see also “Phantom map detection accuracy using coarse arrays” section).

**Table 3:** Grand average training time $\overline{T_t}$ and classification time $\overline{T_c}$ of all 400 generated phantom maps using a dense array (100 samples) and two coarse (stimulation) arrays ($3 \times 5$ and $4 \times 6$ actuators, corresponding to simulation pooling sizes of $15 \times 9$ and $7 \times 7$)

Method	$\overline{T_t}$ (ms)	$\overline{T_c}$ (s)	$\overline{T_t}$ (ms)	$\overline{T_c}$ (s)
Dense array				
	OVA		OVO	
RS	35.0	15.9	54.9	33.7
SS	28.6	15.3	47.9	32.7
MP (2 $\times$ 2)	84.2	17.5	79.3	39.6
AL	300	16.1	278	38
	DAG		BT	
RS	54.9	15.8	24.8	5.57
SS	47.9	15.5	19.8	5.30
MP (2 $\times$ 2)	79.3	16.5	48.1	6.05
AL	278	17	239	5.83
Hybrid coarse (stimulation) array, $3 \times 5$ actuators				
	OVA		OVO	
MP $15 \times 9$	952	34.7	356	46.7
	DAG		BT	
	356	35.8	301.9	9.13
Mechanotactile coarse (stimulation) array, $4 \times 6$ actuators				
	OVA		OVO	
MP $7 \times 7$	348.3	25.2	196	45.0
	DAG		BT	
	196	43.5	153	9.79

*RS* random sampling, *SS* systematic sampling, *MP* majority pooling sampling, *AL* active learning, *OVA* one-vs-all, *OVO* one-vs-one, *DAG* direct acyclic graph, *BT* binary tree

## Discussion

The influences of different SVM algorithms and sampling methods on classification accuracies and training and classification time are discussed in this section.

### Performance of different sampling methods

In general, random and systematic sampling produce higher absolute error rate $E_A$ (Fig. 11). The reason is that the two sampling methods can not sample enough representative data points, as illustrated qualitatively in Fig. 8. Unlike majority pooling, which covers a large range of samples, random and systematic sampling only sample 1% of the total data, thus resulting in poor prediction performance. The two methods also produce a high insufficiency error rate $E_I$ (Fig. 11). This means that the two sampling methods cannot fully grasp the trend of a phantom map model.

All four decomposition multi-class SVM algorithms benefit to various degrees from majority pooling sampling (Fig. 11), which results in more training data sets without increasing the active time involving an amputee. For all algorithms, majority pooling sampling reduces absolute error rate ($E_A$) and functional error rate ($E_F$). It was also observed that for the chosen dense array settings (Table 2), $2 \times 2$ majority pooling produces the smallest error rates for all five error rate metrics.

However when using majority pooling in other settings, there is a trade-off between the pooling-induced error rate and sampling range. A larger pooling size can produce a larger sampling range coverage, but it introduces more pooling-induced errors or noise. For a particular setting, an optimal pooling size exists.

### The influence of different decomposition SVM methods

Overall, OVO-SVM has the smallest absolute error rate (Fig. 11) among the four tested algorithms and the predicted phantom map shapes best represent the original generated phantom map shapes (Fig. 9). The OVO architecture reduces the unclassified regions (compared to the OVA architecture), is less sensitive to unbalanced data sets (compared to the BT architecture), and provides a more thorough evaluation (compared to the DAG architecture).

The error rates of OVA-SVM when using majority pooling are higher than those of OVO-SVM, and lower than those of BT-SVM. The major issue with OVA-SVM is the presence of unclassified regions, as can be seen in Fig. 9b (dashed black lines running within a phantom map finger).

Among the four proposed algorithms, BT-SVM is intrinsically different from the other three multi-class SVMs. All the other three methods classify, to some degree, one class at a time, whereas a BT-SVM tries to separate a group of classes from another group of classes. When classifying a complete phantom map, the BT-SVM first classifies between class group (0, 1, 2) and class group (3, 4, 5). When then classifying between classes (3, 4, 5), it does not consider the classes (0, 1, 2), but only looks for the largest margin between the classes (3, 4, 5) themselves. This can result in two of the class regions being connected in the predicted phantom map, such as in Fig. 9a.

When using BT-SVM, different tree structures can produce different prediction results, especially when dealing with unbalanced data sets. BT-SVM should theoretically have a faster training and classification speed, however, the fast speed is at a price of degraded performance [45].

### The influence of fuzzy logic and active learning

Fuzzy logic and active learning are applied to each decomposition SVM algorithm. A FSVM assigns a fuzzy membership function (8) to each training data set, so that each training data set makes a different contribution in the training process. A FSVM can reduce the influence of pooling induced errors $\overline{E_{\text {MP}}}$ (3). Using FSVMs generally increases the detection accuracy when using the $100 \times 100$ dense arrays. It was also observed that FSVMs reduce the unclassified region for OVA- and OVO-SVM, as was also reported in previous literature [46, 47]. Active learning also helps to increase the detection accuracy by selecting more representative training data.

### Detection accuracy using coarse arrays

Comparing Figs. 11 and 14, it can be observed that the accuracy decreases significantly when using coarse stimulation arrays.

The discussion about the influence of decomposition methods and sampling methods when using a dense array stands true for the coarse array. However, FSVMs do not decreases the error rate when coarse arrays are used. The reason could be that the pooling sizes of coarse arrays are much larger but the sampling density is much smaller, so that the pooling induced error $E_{\text {MP}}$ is larger than that of a dense array.

In order to accurately detect a phantom map distribution, a dense array is therefore needed. However, to the best of our knowledge, no wearable dense array (100 $\times$ 100) is readily available. We therefore propose to divide the design of a sensory feedback system into two parts: the first part makes use of a non-wearable dense array to detect the accurate boundaries of a phantom map, then according to the shape distribution, a customized stimulation array with 20–30 actuators can be integrated into a wearable socket.

### Systematic error caused by socket shifting

As could be expected, all the error rates increase with the shifting degree (Fig. 17). The absolute error rate $E_A$ increases dramatically when the shifting degree increases (Fig. 17). However, the corresponding increase in the average functional error rate $E_F$ is important in relative terms (from 0.12 to 0.97%), still small in absolute terms. The $E_A$ increase at 5% lateral shifting, for example, is indeed mainly caused by an increase of the redundancy error rate (reaching 10.1%) and of the insufficiency error rate $E_I$ (reaching 9.94%).

In other words, despite the fact that $\overline{E_A} = {21.0}{\%}$ at 5% lateral shift, the more critical error rate $\overline{E_F}$ amounts to less than 1%, which is small enough to be negligible. We can conclude that the function of a stimulation array would be minimally affected by a slight socket misalignment.

### Timing

The training and classification times increase substantially with the pooling size, but still stays within an acceptable range. Given the same number of training data sets, the training and classification time were influenced by decomposition architectures. OVO and DAG share the same training process. Under the same conditions, the training times of OVO and DAG are the same. As mentioned in “One-vs-one support vector machine (OVO-SVM)” section, given the same number of training data sets, OVO and DAG-SVM do not require significantly more training time than the other two methods when using random and systematic sampling, and sometimes even less training time when using majority pooling sampling. The classification processes of OVO and DAG-SVM are different. DAG-SVM requires much less classification time than OVO-SVM at the price of a slightly higher absolute error rate ($E_A$). For active learning, due to the iterative nature, the training time was several times longer than that of standard SVMs and FSVMs, although still less than 1 s (Table 3).

## Conclusion

In this paper, three sampling methods and four decomposition multi-class SVMs were proposed for use in automatic phantom map detection.

In the absence of wearable dense stimulation arrays, the accuracy and timing aspects were tested on realistic and flexible generated phantom maps as well as five reported phantom map images and transformations thereof. The phantom map generation algorithm considered different types of phantom maps and introduced parameters to provide a variety of reasonable and representative shapes. The trends of the classification results obtained by the two types of phantom maps are similar. Therefore, the analyses and discussion were applicable for both generated and reported phantom maps.

For the dense stimulation array, we have found that fuzzy OVO-SVM with $2 \times 2$ pooling size has the highest classification accuracy and near real-time training speed (less than 1 s training time for both generated and reported phantom maps).

The potential performance using coarse stimulation arrays, designed primarily to provide sensory feedback, was also evaluated and found to be much lower than that of a dense array. Thus, they are unsuitable for refined phantom map shape detection. We therefore propose a two-step approach, using first a non-wearable dense array to detect an accurate phantom map shape, then to apply a wearable coarse stimulation array customized according to the detection results.

To the best of our knowledge, this is the first attempt to apply machine learning algorithms to identify the distribution of phantom maps. The proposed methodology can be used as a tool for helping haptic feedback designers and for tracking the evolvement of phantom maps.

## Appendix A: Radial basis function and SVM parameter tuning

Kernel functions are essential to expand the classification capacity of a linear classifier to handle non-linear data. In this paper, a radial basis function (RBF) kernel

14 $$\begin{aligned} K(p_i,p_j) ={ \text {exp}} \left (- \frac{ ||p_i-p_j|| ^2}{2 \gamma ^2}\right) \end{aligned}$$

is chosen, because it maps the input space into Hilbert space (an infinite hyperplane) and provides more flexibility [32]. In (14), $p_i$ and $p_j$ are the features from the $i^{th}$ and $j^{th}$ training or classification data sets.

In general, the penalty parameter *C* defines the ‘softness’ of a SVM. A large *C* gives a high penalty for non-separable points. It tends to overfit, exaggerating minor fluctuations or noise in the data. On the other hand, if *C* is too small, the SVM tends to underfit, meaning that the SVM is not able to find the main trend of the underlying data. The RBF kernel parameter $\gamma$ defines the influence of a single training data set: a large $\gamma$ value implies that a single example exerts great influence on the whole SVM model. Both overfitted SVMs and underfitted SVMs produce poor prediction performance.

## Abbreviations

SVM: support vector machine
RS: random sampling
SS: systematic sampling
MPS: majority pooling sampling
RBF: radial basis function
OVA: one-vs-all
OVO: one-vs-one
DAG: directed acyclic graph
BT: binary tree
FSVM: fuzzy support vector machine
